# Pathogenesis of Penile Squamous Cell Carcinoma: Molecular Update and Systematic Review

**DOI:** 10.3390/ijms23010251

**Published:** 2021-12-27

**Authors:** Inmaculada Ribera-Cortada, José Guerrero-Pineda, Isabel Trias, Luis Veloza, Adriana Garcia, Lorena Marimon, Sherley Diaz-Mercedes, José Ramon Alamo, Maria Teresa Rodrigo-Calvo, Naiara Vega, Ricardo López del Campo, Rafael Parra-Medina, Tarek Ajami, Antonio Martínez, Oscar Reig, Maria J. Ribal, Juan Manuel Corral-Molina, Pedro Jares, Jaume Ordi, Natalia Rakislova

**Affiliations:** 1 Department of Pathology, Hospital Clínic of Barcelona, University of Barcelona, 08036 Barcelona, Spain; itribera@clinic.cat (I.R.-C.); jaguerrero@clinic.cat (J.G.-P.); itrias@clinic.cat (I.T.); apgarcia@clinic.cat (A.G.); lorena.marimon@isglobal.org (L.M.); shdiaz@clinic.cat (S.D.-M.); alamo@clinic.cat (J.R.A.); mtrodrigo@clinic.cat (M.T.R.-C.); nvega@clinic.cat (N.V.); rilopez@clinic.cat (R.L.d.C.); antonmar@clinic.cat (A.M.); pjares@clinic.cat (P.J.); jordi@clinic.cat (J.O.); 2 Institute of Pathology, Lausanne University Hospital, Lausanne University, 1011 Lausanne, Switzerland; lveloza@clinic.cat; 3 Barcelona Institute for Global Health (ISGlobal), Hospital Clínic-Universitat de Barcelona, 08036 Barcelona, Spain; 4 Department of Pathology, Research Institute, Fundación Universitaria de Ciencias de la Salud, Bogota 111221, Colombia; rafa.parram@gmail.com; 5 Uro-Oncology Unit, Hospital Clínic of Barcelona, University of Barcelona, 08036 Barcelona, Spain; AJAMI@clinic.cat (T.A.); mjribal@clinic.cat (M.J.R.); jmcorral@clinic.cat (J.M.C.-M.); 6 Institut d’Investigacions Biomèdiques August Pi i Sunyer (IDIBAPS), 08036 Barcelona, Spain; oreig@clinic.cat; 7 Institute of Hematologic and Oncologic Diseases, Hospital Clínic of Barcelona, University of Barcelona, 08036 Barcelona, Spain

**Keywords:** genomic landscape, molecular analysis, next generation sequencing, penile cancer, penile squamous cell carcinoma, whole-exome sequencing, HPV

## Abstract

Penile squamous cell carcinoma (PSCC) is a rare but aggressive neoplasm with dual pathogenesis (human papillomavirus (HPV)-associated and HPV-independent). The development of targeted treatment is hindered by poor knowledge of the molecular landscape of PSCC. We performed a thorough review of genetic alterations of PSCC focused on somatic mutations and/or copy number alterations. A total of seven articles have been identified which, overall, include 268 PSCC. However, the series are heterogeneous regarding methodologies employed for DNA sequencing and HPV detection together with HPV prevalence, and include, in general, a limited number of cases, which results in markedly different findings. Reported top-ranked mutations involve *TP53*, *CDKN2A*, *FAT1*, *NOTCH-1* and *PIK3CA*. Numerical alterations involve gains in *MYC* and *EGFR*, as well as amplifications in HPV integration loci. A few genes including *TP53*, *CDKN2A*, *PIK3CA* and *CCND1* harbor both somatic mutations and copy number alterations. Notch, RTK-RAS and Hippo pathways are frequently deregulated. Nevertheless, the relevance of the identified alterations, their role in signaling pathways or their association with HPV status remain elusive. Combined targeting of different pathways might represent a valid therapeutic approach in PSCC. This work calls for large-scale sequencing studies with robust HPV testing to improve the genomic understanding of PSCC.

## 1. Introduction

Malignant tumors of the penis are rare but impose a major challenge due to their high morbidity and mortality [1]. They occur predominantly in elderly men and their frequency increases with age, reaching its peak between the sixth and the seventh decades of life [2]. Low-income countries in South America and Africa register the highest incidences of penile squamous cell carcinoma (PSCC) [1]. PSCC accounts for around 95% of all malignancies of this organ [3]. The tumor originates most commonly from the epithelium of the glans, inner prepuce and coronal sulcus [4].

**Figure 1:**
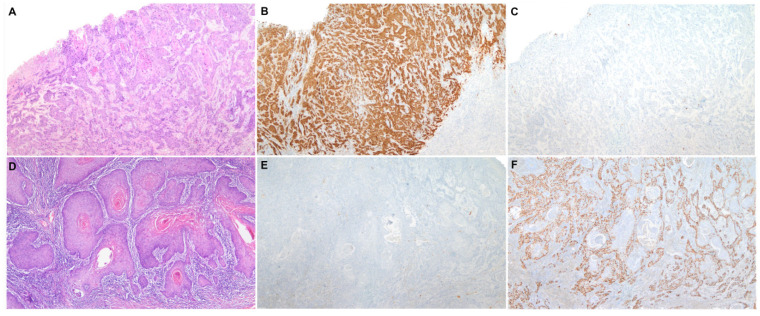
A characteristic example of each of the two types of penile squamous cell carcinoma, HPV-associated and HPV-independent. (**A**) Penile squamous cell carcinoma (H&E 40×) with positive p16 (**B**) and wild-type p53 immunohistochemical stainings (**C**) (40×); (**D**) Penile squamous cell carcinoma (H&E 40×) with negative p16 (**E**) and mutated pattern (diffuse overexpression) of p53 immunohistochemical stainings (**F**) (40×).

Two different etiopathogenic pathways have been described in PSCC [2]: one associated with human papillomavirus (HPV) and the other one independent of this infection. HPV-associated PSCC is more prevalent in relatively young males, who commonly refer to a high number of sexual partners and smoking history [3]. HPV-associated PSCC shows frequently basaloid or warty features and develops on high-grade squamous intraepithelial lesions (HSIL), also known as HPV-associated penile intraepithelial neoplasia (PeIN), Bowen disease or erythroplasia of Queyrat [3]. Immunohistochemical (IHC) overexpression of the p16 protein has been shown to be an accurate surrogate marker of HPV-associated PSCC [4], similar to squamous cell carcinomas of other anatomical sites of the anogenital tract and head and neck region [5]. HPV-independent PSCC is predominant in high-income countries and affects mainly older men [6]. The etiopathogenesis of HPV-independent PSCC is less well understood; however, phimosis, chronic inflammation, poor personal hygiene and trauma seem to be associated factors [3]. Histologically, these tumors are frequently keratinizing and develop from a special type of precursor lesion known as differentiated PeIN (dPeIN) [7]. Both HPV-independent PSCC and its precursor, dPEIN, are almost always negative for p16 [8,9] and frequently show p53 overexpression by IHC [9]. Figure 1 shows a characteristic example of each of the two types of PSCC, HPV-associated and HPV-independent, including the histological features, as well as the p16 and p53 IHC typical patterns of staining. Due to these remarkable epidemiological and clinico-pathological differences, the International Society of Urological Pathology (ISUP) modified, in 2016, its World Health Organization (WHO) classification and categorized PSCCs based on their HPV status and not only on pure histological features [10]. However, in contrast with other anatomical sites where HPV-associated tumors show better prognosis than HPV-independent carcinomas, it remains unclear whether HPV-associated PSCC has a better outcome [11]. Moreover, there are no differences in treatment based on HPV status to date.

Patients with PSCC frequently develop early loco-regional and angiolymphatic spread and have a devastating prognosis [1]. The management of lymph node-negative disease is primarily dependent on risk stratification based on clinico-pathological parameters [12], whereas the management of advanced disease is hampered by partial and short-term response to chemotherapy [13]. These current limitations highlight the need for novel therapeutic options. Regrettably, the low tumor mutation burden [14] and the rare *CD274* (*PD-L1*) amplifications observed in PSCC [15] hint at low responsiveness to immunotherapy [16]. The development of novel biomarkers and therapeutic options is hampered by a limited knowledge of the genomic landscape of PSCC. Remarkably, most of the genetic studies focus on the analysis of single genes (mainly *TP53*, *CDKN2A*, *EGFR*, *PIK3CA* and *MYC*) or analyze a limited number of samples and systematic and extensive genomic analyses of PSCC have not taken place yet.

Since 2014, increased access to next generation sequencing (NGS) has pushed forward the molecular characterization of prevalent neoplasms such as breast or lung cancer. Unfortunately, molecular progress on rare cancers such as PSCC or vulvar cancer [17] has been much slower. We undertook this review to summarize and discuss the findings of the available studies on the genomic landscape of PSCC.

## 2. Methodology

### 2.1. Literature Revision and Criteria of Selection

Relevant studies on genomic alterations (somatic mutations and/or copy number alterations) were captured through a search of Pubmed Medline database using the terms “penis”, “penile”, “cancer”, “carcinoma”, “molecular”, “genomic”, “genetic” and “mutation”. We also conducted a manual search of reference lists from the identified papers. Those original articles focused on genome sequencing in PSCC published until 30 June 2021 and having openly published and extractable datasets were deemed eligible. 

After the first search results, we excluded papers in languages other than English and those with unavailable full text. Then, we screened titles and abstracts excluding misclassified non-original studies (reviews, meta-analyses, editorials or comments), those not focused on human PSCC or not involving DNA sequencing, and other types of documents (books, congress abstracts). After reviewing the full text and examining methodology, we discarded studies not focused on genomic sequencing of PSCC and those assessing less than 10 genes or using non-tissue samples. The results of the eligible studies (tables, figures and Supplementary Materials) were further screened in terms of availability and completeness for each studied gene. 

The researchers extracted the relevant data including the number of PSCC samples and patients studied, the type of DNA sequencing method, the number and prevalence of genomic alterations and the type and results of HPV testing. The genes involved in copy number alterations were searched among those with somatic mutations to identify genes with both types of abnormalities.

### 2.2. Study Selection

**Figure 2:**
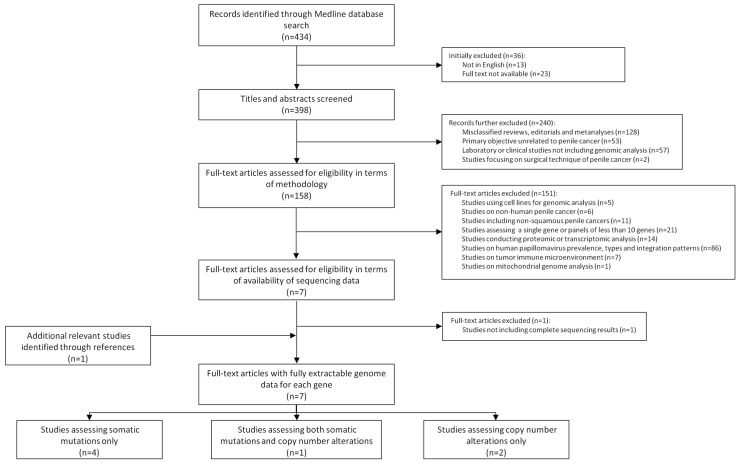
Flowchart with outline of search results and study selection process.

The flowchart of the study with the outline of the search results and the study selection process is shown in Figure 2. The search in Medline Pubmed (accessed on 30 June 2021) rendered 434 articles. Of these, 398 were English-written articles with available full text. After discarding non-original articles, those not focused on human penile cancer and those not applying genomic analysis, 152 studies were further evaluated. More than half of them (86; 57%) were excluded due to the primary focus on HPV prevalence, genotyping or viral integration patterns. Sixty-six articles (43%) were excluded due to the inclusion of non-squamous penile carcinomas, their primary focus on transcriptomic or proteomic analysis or because of a limited number of assessed genes. After evaluating the DNA sequencing results of seven selected articles and reviewing the list of references, one study was not included in the analysis due to the impossibility of reliably extracting the exact numbers from the genomic results [15], while an additional study was captured from the reference list and included in the analysis. 

### 2.3. Methodological Features of the Studies

**Figure 3:**
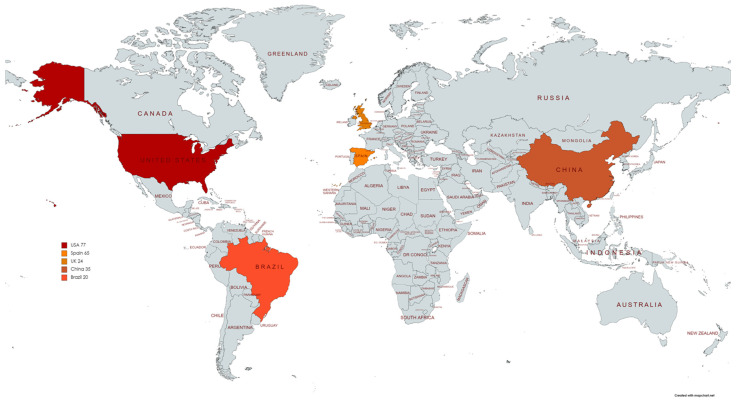
Geographical distribution of the selected study series and the number of patients from each country involved.

Table 1 summarizes the main methodological features and HPV testing results of the seven included study series. Adding all cases reported in the seven series included in the analysis, a total of 268 PSCC samples from 251 patients were analyzed. The studies were published between 2015 and 2021. Two studies (29%) were conducted in the United States, two (29%) in China, one (14%) in the United Kingdom, one (14%) in Brazil and one (14%) in Spain. The geographical distribution of the selected study series is shown in Figure 3.

Four studies (57%) [18,19,20,21] evaluated only somatic mutations, two (29%) [22,23] assessed only copy number profiling and one (14%) [24] included assessment of both somatic mutations and copy number alterations. NGS was applied in five studies (72%), four of them used whole exome sequencing (WES) analysis and one applied a targeted approach using a commercial panel. Three out of the four WES series focused on somatic mutations [18,19,20] and one [23] on copy number alterations. Two studies used other, non-NGS-based methods of genomic analysis: array comparative genomic hybridization [22] and mass spectrometry-based DNA sequencing [21]. One study [24] assessed a subset of matched primary/metastatic tissue. Two studies [20,24] compared their findings of PSCC with those of other types of squamous cell carcinomas using The Cancer Genome Atlas. Four studies, all of them performing WES, analyzed the implicated pathways. IHC or gene expression analysis to validate identified mutations in tissue was conducted in four studies: one used IHC alone [24], one both IHC and Western-Blot [19], one both IHC and PCR [22] and one used only PCR [21]. 

Four of the five NGS studies included an analysis of non-tumor samples, most commonly blood [19,23] or normal penile tissue [20]. The largest WES series included 35 PSCC cases and was focused on copy number DNA analysis. The mean sequencing coverage depth of the WES studies in the tumor samples ranged from 60× [18] to 141× [20], whereas the targeted NGS study [24] was sequenced at 535x. Three out of four WES studies [18,19,20] additionally explored mutational signatures.

HPV testing based on PCR was performed in all seven studies, however, only five correlated HPV status and molecular results. The HPV tests included PCR-reverse dot blot assay (two series), unspecified PCR (two series), PGMY9/11 (two series) and Cobas HPV assay (one study). Only three studies, including one of the WES series [20], additionally conducted p16 IHC. The HPV positivity rates ranged from 12% in the American cohort [24] to 96% in the Brazilian study [22]. 

Four out of seven studies (57%) evaluated the prognostic implications of the genomic alterations identified in PSCC. The follow-up ranged from 27 [21] to 96 months [24]. 

## 3. Results

### 3.1. Somatic Mutations 

Table 2 features the results of the five studies [18,19,20,21,24] on somatic mutations in PSCC. Top-ranked somatic mutations comprised *TP53*, *CDKN2A*, *NOTCH1*, *PIK3CA*, *FAT1*, *CASP8* and *FBXW7*. *TP53* was mutated in 32% (48/148) of the assessed samples, with frequencies ranging from 10 to 48%. Strikingly, few recurrent somatic alterations were reported in the two WES series (17% and 11%) [18,19]. 

### 3.2. Copy Number Variations

Table 3 shows the results of the three studies on copy number variations in PSCC. The chromosome region analyzed, the type of event detected, the targeted genes, the total number of tumors analyzed, the number of tumors showing alterations in each region and the overall frequency and range of alterations are shown. The most common copy number variations included gains in 8q24 (*MYC* locus). Two studies identified copy number variations involving the locus of *EGFR* in 10 to 70% of cases [22,24]. The Brazilian series [22] showed correlation of *EGFR* variations with increased tumor size. McDaniel et al. [24] also showed high heterogeneity in copy number variations between matched primary tumors and metastasis by finding only 42% of concordance.

Amplifications or gains at HPV integration sites 14q32.33 (loci of noncoding RNAs (*IncRNAs*, *ADAM6*, *LINC00226*, *LINC00221* and *KIAA0125*), 2p12-p11.2, 10q26.13 and 8q23.1 were identified with high frequencies (85–100%); however, all of them were reported in a single study [22]. Somatic mutations, in addition to copy number alterations, have been reported in a few genes including *TP53*, *CDKN2A*, *PIK3CA*, *CCND1*, *ALK*, *BIRC6*, *IL7R*, *PDE4DIP* and *LAMA1*. 

### 3.3. Relationship with HPV Status 

Among the five studies that have compared the genomic alterations identified in HPV-associated and HPV-independent PSCC, two [18,24] reported a markedly lower mutational load (number of non-silent and driver mutations) in HPV-associated than in HPV-independent PSCC. Contrarily, a WES study [20] found only minimal, negligible mutation load differences between the two etiopathogenic types. Two studies [20,24] identified a strong inverse correlation between HPV positivity and *TP53* and *CDKN2A* mutations. In one of them [20], HPV-associated tumors were significantly associated with somatic mutations in *ARPP21*, *CMYA5*, *RPGRIP* and *CSPG4*.

Regarding copy number alterations, one study [24] showed low frequency in HPV-associated PSCC, whereas the Brazilian series, with 95% of the tumors being HPV-associated [22], reported high rates of copy number alterations in HPV integration sites (2p12-p11.2 and 14q32.33), as well as in inflammation-related genes (*EGFR* and *COX2*). 

### 3.4. Mutational Signatures and Signaling Pathways in PSCC

Transition mutations, including mostly C>T alterations, mediated by the APOBEC family of cytosine deaminases [25] were reported in three studies [18,19,20]. Feber et al. [18] identified HPV-associated APOBEC mutation signatures and NpCgP signatures in HPV-negative PSCC, in which C>T alterations correlated with decreased DNA methylation. Chahoud et al. [20] additionally reported a subset of PSCC with a defective DNA repair system (*BRCA1*, *BRCA2*, *ARID1A*, *ATR*, *CHEK2*, *PARP1*, *FANCA*, *PALB2*, and *RAD51*). 

At least 10 signaling pathways have been identified as disrupted in PSCC in four WES studies. Of them, two [19,20] (50%) reported the Notch, RTK-RAS and Hippo signaling pathways as the three most implicated. The WES study conducted in China [19] showed alterations in these three pathways in more than half of the samples. By contrast, McDaniel et al. [24] showed the predominance of the p53 pathway deregulation. On the other hand, one of the studies on copy number variants [23] highlighted the role of the MYCN/Max pathway. 

### 3.5. Prognostic Implications of Mutations and Copy Number Alterations in PSCC

McDaniel et al. [24] noticed that patients with *MYC* and *CCND1* gains, and those with negative p16 IHC, showed shorter time to progression or survival. The same study reported that high mutational burden in the five most frequently mutated genes (*CDKN2A*, *EGFR*, *MYC*, *HRAS* and *TP53*) correlated with an advanced stage.

Amplifications in *MYCN* and *FAK* correlated significantly with worse survival in one study [23]. Chahoud et al. [20] showed a trend towards worse overall survival for patients with mutations in the Notch pathway, whereas patients with the PI3K pathway mutated genes had improved outcomes. High APOBEC scores correlated with shorter overall survival, higher tumor mutational burden and the presence of lymph node metastasis [20]. 

### 3.6. Potential Therapeutic Targets to Treat PSCC

Most of the studies suggest potential actionable targets on the basis of the identified genomic alterations. McDaniel et al. [24] proposed targeting amplifications of *EGFR* and cell cycle kinase *CDK4*, as well as somatic mutations in *KRAS*. Ferrándiz-Pulido et al. [21] indicated that patients with concomitant KRAS and PIK3CA mutations might benefit from a combination of mTOR and tyrosine-kinase inhibitors. The same authors proposed imatinib for patients with *PDGFA*-mutated tumors. Chahoud et al. [20] hypothesized that patients with APOBEC-enriched tumors might benefit from immune checkpoint inhibitors, whereas those with a defective DNA repair system and microsatellite instability might be treated with PARP inhibitors alone or combined with immune checkpoint inhibition. The same study provided an extensive list of druggable genes using the Drug Gene Interaction database, which included genes such as *TP53*, *CDKN2A*, *NOTCH1*, *PIK3CA*, *FBXW7*, *CASP8*, *LAMA1* and *TTN*, among others. 

The Brazilian series, which mostly included HPV-positive tumors [22], proposed targeting *ADAM6* alterations involved in the Notch pathway, although there is little knowledge of their role in cancer. The same authors proposed using *EGFR* and *COX2* inhibitors. 

## 4. Discussion

Considerable insight on the genomic landscape of PSCC has been acquired over the last six years (2015–2021), as evidenced by the seven studies identified. However, these studies are highly heterogeneous in terms of sociodemographic characteristics, methodology (tissue analyzed, frozen or paraffinized, type of genomic analysis) and include a limited number of samples, which may hamper the validity of some of the conclusions. As a result, the series are also highly heterogeneous in terms of their findings. 

Somatic mutations in cancer-related genes *TP53*, *CDKN2A*, *FAT1*, *NOTCH1* and *PIK3CA* are consistently identified in PSCC. Copy number alterations have also been reported in three of these genes (*TP53*, *CDKN2A* and *PIK3CA*) [20,21], which speaks to the relevance of these genes in PSCC carcinogenesis. *TP53* and *CDKN2A* are well-known tumor suppressor genes [15,20,24]. *NOTCH1* and *FAT1* mutations are consistently featured in PSCC [15]. However, little is known on the role and mechanisms of both types of mutations in PSCC and other cancers [26]. 

Another intriguing and frequent finding of the recent studies [18,19,20] includes the identification of *CASP8* and *FBXW7* alterations. *CASP8* is known for its involvement in apoptosis cascade, whereas *FBXW7* acts as a promoter of tumorigenesis through ubiquitin degradation of cell cycle regulators, including p53 [27]. As occurs with *FAT1* mutations, the contribution and clinical relevance of both genes in PSCC remain to be elucidated. Curiously, patients with *TP53* wild-type tumors of oral cavity harboring both *CASP8* and *HRAS* mutations showed improved outcomes [28]. It is also interesting to further explore the role of the *NBPF1* gene in PSCC, identified with high frequency but only in a single study. *NBPF1* is known to deactivate the PI3K signaling pathway leading to tumor growth inhibition [29]. 

The genomic profile of PSCC also typically contains numerical alterations in *MYC*, *EGFR* and *CCND1*. The high numbers of *MYC* and *EGFR* variations in PSCC are in concordance with previous evidence reported in head and neck cancers squamous cell carcinoma, a similar tumor with dual pathogenesis [30]. 

Notch represents the most involved signaling pathway in the studies exploring the whole exome. Curiously, the PI3K pathway is not among the three most frequently involved signaling pathways, despite frequent identification of *PIK3CA* and *EGFR* alterations [19,20]. It is of note, however, that both genes are also implicated in the Hippo pathway, among the three most implicated pathways in this review.

Although PSCC has been divided into two different etiologic pathways (HPV-associated and -independent), the overall mutational profile of HPV-associated PSCC is not considerably different from HPV-independent tumors in the published studies. However, a marked variability in HPV prevalence hampers comparability of findings among studies. Indeed, whereas the HPV prevalence ranged from 12 to 37 in six of the studies [18,19,20,23,24], which is in keeping with the numbers reported in most studies on PSCC [15,31,32], one of the series [22] reports an unusually high percentage (96%) of HPV-associated tumors. 

The prognostic role of most molecular alterations in PSCC also remains elusive. Remarkably, only a single study in this review [20], based on WES, finds prognostic association for PI3K pathway mutations, *NOTCH1* mutations or APOBEC scores. The association of MYC gains with adverse prognosis was also shown in a single study [24], in accordance with only one available publication [33]. The prognostic relevance of *MYCN* and *FAK* variations described by Yongbo et al. [23] certainly warrant further research using a similar approach based on WES. 

Unfortunately, the genes most frequently altered in PSCC, including *TP53*, *CDKN2A*, *PIK3CA*, *MYC*, and *EGFR*, have proven to be challenging to target separately [34,35,36]. Thus, it might be worth exploring combinations of treatments based on an interaction between implicated signaling pathways. For example, mutant p53 is highly oncogenic through the stimulation of the PI3K signaling pathway, which suggests the utility of mTOR inhibitors in *TP53*-mutated patients [37]. Patients with defective DNA repair and APOBEC systems might respond to PARP and checkpoint immune inhibition [20]. Lastly, since both *NOTCH1* and *PIK3CA* mutations are frequent in PSCC [20], vulvar [17] and head and neck squamous cell tumors [38], there is rationale to enroll such patients in clinical trials focused on PI3K/mTOR inhibitors in *NOTCH1*-mutated patients.

High heterogeneity in findings among the studies might be due to methodological differences in DNA sequencing. Indeed, the targeted NGS study [24], which explores 126 genes, prioritizing recurrently altered and tumor suppressor genes, cannot be compared with WES studies covering around 20,000 genes. Nevertheless, even the three WES studies are heterogenous in terms of results and methods. The low number of mutations (only 12 genes) reported by the Chinese WES study [19], in contrast with at least double the number of mutations detected in the other WES series, is striking [18,20]. Indeed, while the earliest WES study [18] reports 60x coverage using Hi-Seq2000, the most recent WES series [19,20] use a more advanced (Hi-Seq2500 or Hi-Seq4000) sequencer, with coverages ranging from 130x to 141x. 

In conclusion, there is still limited understanding of molecular abnormalities involved in PSCC. There is a lack of evidence regarding the association of molecular abnormalities with main clinico-pathological variables. The existing studies are limited in sample size, sociodemographic heterogeneity and variability in DNA sequencing methodology. There is a particular gap of knowledge in the characterization of molecular profiles in relation to HPV status. Given the rarity of PSCC, especially in high-income countries, a number of genomic studies regarding this disease face challenges in acquiring enough samples. Therefore, large multicenter studies are urgently needed to continue on the path of the molecular characterization of PSCC. 

## Figures and Tables

**Table 1:** Main characteristics of the studies analyzing the genomic alterations in Penile Squamous Cell Carcinoma.

Author, Year and Reference	Country	Number of Patients	Number of Samples	Characteristics of the Sample	HPV Test	HPV Prevalence	Type of Genomic Analysis	Gene Panel	Number of Targeted Genes	Platform/Sequencing Depth (for NGS Studies)
**Studies assessing only somatic mutations (*n* = 4)**										
Ferrándiz-Pulido (2015)	Spain	65	65	FFPE	Unspecified PCR and p16	28%	Targeted mass spectrometry sequencing	Oncocarta mutation panel v1.0	19	N/A
Feber (2016)	UK	24	24	Not specified	Unspecified qPCR	37%	Whole exome sequencing	N/A	Whole exome	Hi-Seq 2000/60x
Wang (2019)	China	30	30	Fresh frozen	PCR-reverse dot blot assay	20%	Whole exome sequencing	N/A	Whole exome	Hi-Seq 2500/130x
Chahoud (2021)	USA	34	34	Fresh frozen	Cobas HPV assay and p16	29%	Whole exome sequencing	N/A	Whole exome	Hi-Seq 4000/141x
**Studies assessing both somatic mutations and copy number variations (*n* = 1)**										
McDaniel (2015)	USA	43	60 *	FFPE	GP5+/GP6+My09/11 and p16	12%	Multiplex-based targeted NGS	Oncomine Comprehensive Panel	126	PGM/535x
**Studies assessing only copy number variations (*n* = 2)**										
Macedo (2020)	Brazil	20	20	FFPE and fresh frozen	My09/My11	96%	aCGH; TaqMan copy number assay in the genes of PI3K/AKT pathway	N/A	N/A	N/A
Yongbo (2020)	China	35	35	Fresh frozen	PCR-reverse dot blot assay	20% **	Whole exome sequencing	N/A	Whole exome	Hi-Seq2500/120x

* A subset of matched primary/metastatic tissue was assessed; ** frequency based on 30 out of 35 samples; aCGH: comparative genomic hybridization; FFPE: formalin fixed paraffin embedded; N/A: not applicable; HPV: human papillomavirus; NGS: next generation sequencing; PCR: polymerase chain reaction; UK: United Kingdom; USA: United States of America.

**Table 2:** Frequencies of somatic mutations identified in individual genes, stratified by most frequently altered genes, in penile squamous cell carcinomas (PSCC).

Gene	Studies Evaluating the Gene	Studies Identifying Alterations in the Gene	Total Number of PSCC Assessed	Number of PSCC with Alterations in the Gene	Overall Frequency (%)	Frequency Range (%)
**Genes identified in more than one study**						
*TP53*	4	4	148	48	32.4	10–48
*NOTCH1*	4	4	148	26	17.6	7–44
*PIK3CA*	4	4	189	25	13.2	9–21
*HRAS*	4	4	179	20	11.2	6–17
*CDKN2A*	3	3	118	30	25.4	4–32
*FAT1*	3	3	88	22	25.0	13–35
*CASP8*	3	3	88	15	17.0	13–24
*FBXW7*	3	3	118	13	11.0	8–15
*NFE2L2*	3	3	114	12	10.5	8–12
*TTN*	2	2	64	14	21.9	10–32
*MUC17*	2	2	58	8	13.8	13–15
*FLG*	2	2	54	7	12.9	10–17
*EP300*	2	2	58	6	10.3	4–15
*KRAS*	2	2	125	8	6.4	3–9
*KIT*	2	2	89	4	4.5	3–8
*BRAF*	2	2	125	4	3.0	3–3
**Genes identified in a single study**						
*NBPF1*	1	1	24	13	54.2	N/A
*MLL3*	1	1	24	9	37.5	N/A
*HLA-B*	1	1	24	5	20.8	N/A
*MUC4*	1	1	34	7	20.6	N/A
*DNAH6*	1	1	34	6	17.6	N/A
*GXYLT1*	1	1	24	4	16.7	N/A
*AHNAK2*	1	1	34	5	14.7	N/A
*LAMA1*	1	1	34	5	14.7	N/A
*MUC2*	1	1	34	5	14.7	N/A
*XRP2*	1	1	24	3	12.5	N/A
*NSD1*	1	1	24	3	12.5	N/A
*IL7R*	1	1	24	3	12.5	N/A
*DNAH12*	1	1	24	3	12.5	N/A
*WASF3*	1	1	24	3	12.5	N/A
*TSC1*	1	1	24	3	12.5	N/A
*SETDB1*	1	1	24	3	12.5	N/A
*NF1*	1	1	24	3	12.5	N/A
*COL5A3*	1	1	24	3	12.5	N/A
*CHD4*	1	1	24	3	12.5	N/A
*ANK3*	1	1	24	3	12.5	N/A
*ALK*	1	1	24	3	12.5	N/A
*ZNF462*	1	1	24	3	12.5	N/A
*ZBTB5*	1	1	24	3	12.5	N/A
*NID1*	1	1	24	3	12.5	N/A
*IQGAP2*	1	1	24	3	12.5	N/A
*INSR*	1	1	24	3	12.5	N/A
*HEXA*	1	1	24	3	12.5	N/A
*CNTLN*	1	1	24	3	12.5	N/A
*PFAS*	1	1	24	3	12.5	N/A
*PAPLN*	1	1	24	3	12.5	N/A
*CENPJ*	1	1	24	3	12.5	N/A
*C2CD3*	1	1	24	3	12.5	N/A
*ATP10D*	1	1	24	3	12.5	N/A
*ASXL1*	1	1	24	3	12.5	N/A
*HHAT*	1	1	24	3	12.5	N/A
*AK302511*	1	1	34	4	11.8	N/A
*ARPP21*	1	1	34	4	11.8	N/A
*BIRC6*	1	1	34	4	11.8	N/A
*CACNA1C*	1	1	34	4	11.8	N/A
*CSPG4*	1	1	34	4	11.8	N/A
*FAT4*	1	1	34	4	11.8	N/A
*FHAD1*	1	1	34	4	11.8	N/A
*FRG1*	1	1	34	4	11.8	N/A
*FRY*	1	1	34	4	11.8	N/A
*FSIP2*	1	1	34	4	11.8	N/A
*GRIN2B*	1	1	34	4	11.8	N/A
*KMT2B*	1	1	34	4	11.8	N/A
*MYO188*	1	1	34	4	11.8	N/A
*PDE4DIP*	1	1	34	4	11.8	N/A
*PKD1*	1	1	34	4	11.8	N/A
*SLITRK2*	1	1	30	3	10.0	N/A
*TRRAP*	1	1	30	3	10.0	N/A
*CCDC168*	1	1	30	3	10.0	N/A
*SACS*	1	1	24	2	8.3	N/A
*NUP210L*	1	1	24	2	8.3	N/A
*MGA*	1	1	24	2	8.3	N/A
*USP31*	1	1	24	2	8.3	N/A
*TM9SF1*	1	1	24	2	8.3	N/A
*TGM1*	1	1	24	2	8.3	N/A
*SNX19*	1	1	24	2	8.3	N/A
*SMG6*	1	1	24	2	8.3	N/A
*SLC7A6OS*	1	1	24	2	8.3	N/A
*PITPNM2*	1	1	24	2	8.3	N/A
*PIGT*	1	1	24	2	8.3	N/A
*NCF2*	1	1	24	2	8.3	N/A
*MTHFR*	1	1	24	2	8.3	N/A
*IQCG*	1	1	24	2	8.3	N/A
*INADL*	1	1	24	2	8.3	N/A
*GPS1*	1	1	24	2	8.3	N/A
*FAM72D*	1	1	24	2	8.3	N/A
*DFNA5*	1	1	24	2	8.3	N/A
*CX3CR1*	1	1	24	2	8.3	N/A
*CREB3L4*	1	1	24	2	8.3	N/A
*CPNE1*	1	1	24	2	8.3	N/A
*CHPT1*	1	1	24	2	8.3	N/A
*BRCA1*	1	1	24	2	8.3	N/A
*ZFHX3*	1	1	24	2	8.3	N/A
*TXNDC8*	1	1	24	2	8.3	N/A
*TNFRSF14*	1	1	24	2	8.3	N/A
*TGFBR2*	1	1	24	2	8.3	N/A
*TET2*	1	1	24	2	8.3	N/A
*TDRD10*	1	1	24	2	8.3	N/A
*SNX25*	1	1	24	2	8.3	N/A
*SELP*	1	1	24	2	8.3	N/A
*PRDM1*	1	1	24	2	8.3	N/A
*OTUD7A*	1	1	24	2	8.3	N/A
*NTN4*	1	1	24	2	8.3	N/A
*NCOR1*	1	1	24	2	8.3	N/A
*HLA-A*	1	1	24	2	8.3	N/A
*CREBBP*	1	1	24	2	8.3	N/A
*BRE*	1	1	24	2	8.3	N/A
*ATM*	1	1	24	2	8.3	N/A
*PDGFA *	1	1	65	3	4.6	N/A
*ZRANB3*	1	1	24	1	4.2	N/A
*ZNF180*	1	1	24	1	4.2	N/A
*TIMM17A*	1	1	24	1	4.2	N/A
*STK19*	1	1	24	1	4.2	N/A
*SPEN*	1	1	24	1	4.2	N/A
*OR52N1*	1	1	24	1	4.2	N/A
*OR4A16*	1	1	24	1	4.2	N/A
*MYOCD*	1	1	24	1	4.2	N/A
*MORC4*	1	1	24	1	4.2	N/A
*MICALCL*	1	1	24	1	4.2	N/A
*ITPKB*	1	1	24	1	4.2	N/A
*FAM166A*	1	1	24	1	4.2	N/A
*DIS3*	1	1	24	1	4.2	N/A
*CTCF*	1	1	24	1	4.2	N/A
*C3orf70*	1	1	24	1	4.2	N/A
*BCLAF1*	1	1	24	1	4.2	N/A
*ALPK2*	1	1	24	1	4.2	N/A
*NRAS *	1	1	65	2	3.1	N/A
*SMARCB1*	1	1	60	1	1.7	N/A
*ABL *	1	1	65	1	1.5	N/A
*EGFR *	1	1	65	1	1.5	N/A
*MET *	1	1	65	1	1.5	N/A
*RET *	1	1	65	1	1.5	N/A

The most frequent (but not the most studied) somatic mutations were identified in *NBPF1* (13/24; 54%), followed by *MLL3* (9/24; 37.5%). Both mutations were identified in a single WES study [18].

**Table 3:** Frequencies observed in copy number alteration studies of identified alterations in individual genes, stratified by most frequently altered genes, in penile squamous cell carcinomas (PSCC). The genes showing both somatic mutations and copy number alterations are highlighted in bold.

Chromosome Region Studied	Event	Targeted Genes	Studies Identifying Alterations in the Gene	Number of PSCC Assessed	Number of PSCC with Gene Alteration	Overall Frequency (%)	Frequency Range (%)
**Copy number alterations identified in more than one study**							
8q24	Gains	*MYC*	2	80	26	32.5	18–75
7p12.1 to 11.2	Gains	***EGFR***	2	80	20	25.0	10–70
**Copy number alterations identified in one study**							
14q32.33	Amplifications	*ADAM 6, KIAA0125, LINC00226, LINC00221* *miR7641-2*	1	20	20	100	N/A
2p12-p11.2	Gains	*REEP, CTNNA2, LRRTM1, ATOH8, DNAH6, FABP1, CD8A, CD8B, C2orf3, FAM176A, SUCCLG1, ELMOD3, USP39, VAMP8, FOXI3, FAM176A, SMYD1*	1	20	20	100	N/A
10q26.13	Gains	*MGMT, EBF3, JAKMIP3, INPP5A, KNDC1, GLRX3, PPP2R2D, BNIP3, DPYSL4, LRRC27, ADAM8, PRAP1, PTER, PAOX, MTG1, CALY, SPRN, UTF1*	1	20	17	85.0	N/A
8p23.1	Losses	*MCPH1, SGK223, SOX7, GATA4, PINX1, TDH, FAM66A*	1	20	17	85.0	N/A
10q11.22	Losses/deletions	*ZNF488, GDF2, SYT15, MAPK8, RBP3*	1	20	15	75.0	N/A
1p36.3	Gains	*VWA1, CCNL2, MIB2, ATAD3A, GNB1, HES5, TP73*	1	20	15	75.0	N/A
14q11.2	Losses/deletions	*CHD8, TOX4, APEX1, SALL2*	1	20	14	70.0	N/A
15q11.2-q13.3	Gains	*OCA2, CYFIP1, TRPM1, BCL8*	1	20	13	65.0	N/A
8q11.1-q24.3	Gains	*TOX, WISP1, IL7, STK3, SOX17, RP1, MAFA*	1	20	13	65.0	N/A
10p11.23	Deletions	*Bmi1*	1	35	22	62.9	N/A
1q43	Gains	*IRF2BP2, ARID4b, LYST, GGPS1, FMN2*	1	20	12	60.0	N/A
7q21.11	Gains	*CD36, GNAT3, TMEM60, PHTF2*	1	20	12	60.0	N/A
2p24.3	Amplifications	*MYCN*	1	35	20	57.1	N/A
15q11.1-q11.2	Losses/deletions	*BCL8, CYFIP1, NIPA1, NIPA2*	1	20	11	55.0	N/A
17p13.1	Deletions	***TP53***	1	35	19	54.3	N/A
8q24.3	Amplifications	*PTK2 (FAK)*	1	35	19	54.3	N/A
12q15	Deletions	*MDM2*	1	35	18	51.4	N/A
22q11.21	Gains	*BID, CECR2, TBX1, GSC2, HIRA*	1	20	10	50.0	N/A
2p25.3-p11.1	Gains	*SOX11, REL, FOXI3, EGR4, SIX3, TLX2, **BIRC6**, CD8B*	1	20	10	50.0	N/A
3q26.1	Gains	*MECOM, SI, PDCD10*	1	20	10	50.0	N/A
17q21.33	Deletions	*NGFR (p75NTR)*	1	35	17	48.6	N/A
15q26.2-q26.3	Gains	*CHD2, IGF1R, RGMA, MEF2A, PTER, SYNM*	1	20	9	45.0	N/A
4q13.2	Losses	*UGT2B4, STAP1, TMPRSS11D*	1	20	9	45.0	N/A
5q13.2	Losses/deletions	*SKP2, BRIX1, **IL7R**, GDNF, RAD1, BRIX1, SPEF2*	1	20	9	45.0	N/A
7p21.3	Gains	*PHF14, COL281A, RPA3, ARL4A*	1	20	9	45.0	N/A
14q12	Gains	*REC8, TINF2, PRKD1, IL25, FOXG1*	1	20	8	40.0	N/A
16p11.2-p11.1	Gains	*ZNF689, ZNF668, YPEL3, PYCARD, MAZ, IL27, CD19*	1	20	8	40.0	N/A
1q21.2	Losses	*POLR3C, BCL9, **PDE4DIP**, TXNIP, CD160, ACP6*	1	20	8	40.0	N/A
20q13.32-q13.33	Gains	*BIRC7, DIDO1, ZGPAT, CTCFL, GNAS, SPO11, SLC2A4RG, GATA5, TAF4, PTK6*	1	20	8	40.0	N/A
9p21	Gains	*CDKNB, ACO1, TAF1L, TEK, IFT74, IFNK, TUSC1, MLLT3*	1	20	8	40.0	N/A
1q23.1	Amplifications	*NTRK1(TRKA)*	1	35	13	37.1	N/A
2p16.3	Gains	*MSH2, MSH6, FOXN2, NRXN1, FSHR*	1	20	7	35.0	N/A
1p36.13	Deletions	*ZBTB17(Miz1)*	1	35	12	34.3	N/A
11q24-q25	Gains	*ST14, CDON, OPCML, BARX2, ETS1, ADAMTS15, PTER*	1	20	5	25.0	N/A
14q31-q31.3	Gains	*GPR65, STON2, FLRT2*	1	20	5	25.0	N/A
18p11.31 -p11.21	Gains	***LAMA1**, APCDD1, TGIF1, MC5R*	1	20	5	25.0	N/A
7p22.3-p11	Gains	*PMS2, ADAP1, FAM126A, TSPAN13, MAFK*	1	20	5	25.0	N/A
Yp11.3-p11.2	Gains	*CD99, ZBED1, TSPY1*	1	20	5	25.0	N/A
9p21.3	Losses	***CDKN2A***	1	60	13	21.6	N/A
14q23.3	Amplifications	*Max*	1	35	7	20.0	N/A
11q13.3	Gains	***CCND1***	1	60	8	13.3	N/A
3q26.33	Gains	*SOX2*	1	60	8	13.3	N/A
3q26.33	Gains	*ATP11B*	1	60	5	8.3	N/A
5p15.33	Gains	*TERT*	1	60	4	6.7	N/A
3q26.33	Gains	*DCUN1D1*	1	60	3	5.0	N/A
10p14	Losses	*GATA3*	1	60	2	3.3	N/A
11p13	Gains	*CD44*	1	60	2	3.3	N/A
22q12.2	Losses	*NF2*	1	60	2	3.3	N/A
3q26.32	Gains	***PIK3CA***	1	60	2	3.3	N/A
11q22.2	Gains	*BIRC3*	1	60	1	1.7	N/A
12q14.1	Gains	***CDK4***	1	60	1	1.7	N/A
20q11.21	Gains	*BCL2L1*	1	60	1	1.7	N/A
4q31.3	Losses	***FBXW7***	1	60	1	1.7	N/A

N/A: not applicable.
